# Clinical and economic impact of ‘*ROS1-testing’* strategy compared to a *‘no-ROS1-testing’* strategy in advanced NSCLC in Spain

**DOI:** 10.1186/s12885-022-09397-4

**Published:** 2022-03-19

**Authors:** Federico Rojo, Esther Conde, Héctor Torres, Luis Cabezón-Gutiérrez, Dolores Bautista, Inmaculada Ramos, David Carcedo, Natalia Arrabal, J. Francisco García, Raquel Galán, Ernest Nadal

**Affiliations:** 1 Hospital Universitario Fundación Jiménez Diaz – CIBERONC, Madrid, Spain; 2 Hospital Universiario 12 de Octubre-CIBERONC, Madrid, Spain; 3 Hospital Universitario Central de Asturias, Oviedo, Spain; 4 Hospital Universitario de Torrejón, Torrejón De Ardoz, Spain; 5 Hospital Costa del Sol, Marbella, Spain; 6 Hospital Virgen de la Victoria, Málaga, Spain; 7 Hygeia Consulting, S.A, Madrid, Spain; 8 Hygeia Consulting, Barcelona, Spain; 9 Roche Farma S.A, Madrid, Spain; 10 Catalan Institute of Oncology, Hospital Duran i Reynals, IDIBELL, L’Hospitalet de Llobregat, Spain

**Keywords:** C-ros oncogene 1, Non-small cell lung cancer, Molecular testing, Biomarker guided selection, Cost-effectiveness analysis

## Abstract

**Background:**

Detection of the *ROS1* rearrangement is mandatory in patients with advanced or metastatic non-small cell lung cancer (NSCLC) to allow targeted therapy with specific inhibitors. However, in Spanish clinical practice *ROS1* determination is not yet fully widespread. The aim of this study is to determine the clinical and economic impact of sequentially testing *ROS1* in addition to *EGFR* and *ALK* in Spain.

**Methods:**

A joint model (decision-tree and Markov model) was developed to determine the cost-effectiveness of testing *ROS1* strategy versus a no-*ROS1* testing strategy in Spain. Distribution of *ROS1* techniques, rates of testing, positivity, and invalidity of biomarkers included in the analysis (*EGFR*, *ALK*, *ROS1* and PD-L1) were based on expert opinion and *Lungpath* real-world database. Treatment allocation depending on the molecular testing results was defined by expert opinion. For each treatment, a 3-states Markov model was developed, where progression-free survival (PFS) and overall survival (OS) curves were parameterized using exponential extrapolations to model transition of patients among health states. Only medical direct costs were included (€ 2021). A lifetime horizon was considered and a discount rate of 3% was applied for both costs and effects. Both deterministic and probabilistic sensitivity analyses were performed to address uncertainty.

**Results:**

A target population of 8755 patients with advanced NSCLC (non-squamous or never smokers squamous) entered the model. Over a lifetime horizon, the *ROS1* testing scenario produced additional 157.5 life years and 121.3 quality-adjusted life years (QALYs) compared with no-*ROS1* testing scenario. Total direct costs were increased up to € 2,244,737 for *ROS1* testing scenario. The incremental cost-utility ratio (ICUR) was 18,514 €/QALY. Robustness of the base-case results were confirmed by the sensitivity analysis.

**Conclusions:**

Our study shows that *ROS1* testing in addition to *EGFR* and *ALK* is a cost-effective strategy compared to no-*ROS1* testing, and it generates more than 120 QALYs in Spain over a lifetime horizon. Despite the low prevalence of *ROS1* rearrangements in NSCLC patients, the clinical and economic consequences of *ROS1* testing should encourage centers to test all advanced or metastatic NSCLC (non-squamous and never-smoker squamous) patients.

**Supplementary Information:**

The online version contains supplementary material available at 10.1186/s12885-022-09397-4.

## Background

Lung cancer (LC) has a high incidence rate worldwide and is the main cause of cancer deaths (18.0% of all cancer deaths), so it represents a major health problem [1–4]. In Spain, according to the Spanish Society of Medical Oncology (SEOM), in 2020 lung cancer was responsible for the highest number of cancer deaths in Spain, causing 22,930 deaths (20.3% of all cancer deaths) [3].

Non-small cell lung cancer (NSCLC) accounts for 85% of lung cancer cases and is classified into several histological subtypes, of which adenocarcinoma is the most common (55–60% of LC) [5]. In these histological subtypes, a wide variety of oncogenic driver alterations have been described, such as the presence of translocations or rearrangements of the anaplastic lymphoma kinase (*ALK*) gene, mutations in the epidermal growth factor receptor (*EGFR*) gene, rearrangements of the *c-ros oncogene 1* (*ROS1*) gene, and also the presence of aberrant expression of programmed death-ligand 1 (PD-L1) [1]. Specifically, the *ROS1* gene encodes a receptor with tyrosine kinase activity that is altered by chromosomal rearrangement in several tumor types, including LC where it can be detected in approximately 1% of NSCLC patients and appears to be associated with low tobacco exposure and adenocarcinoma histology [1, 6].

Patients with advanced LC generally have a poor prognosis; however, the advent of targeted therapy directed to oncogenic genetic alterations has created a new landscape, especially in NSCLC treatment, providing significant improvements in survival and quality of life [7, 8]. The growing number of targeted therapies to *EGFR* and *ALK* alterations has resulted in a rapid change in the prognostic of these subtype of NSCLC patients [9]. In particular, targeted therapy with specific inhibitors of *ROS1* rearrangements in patients with advanced NSCLC has shown longer overall survival than patients treated with conventional chemotherapy. According to several studies, long-term disease control exerted by crizotinib in patients with *ROS1* rearrangement is almost double that the control obtained in patients with *ALK* alterations [6, 10–13]. In addition, other drugs, such as entrectinib, brigatinib, lorlatinib and ceritinib, are being studied to treat patients harboring *ROS1*-positive cancers [1], but at the time of the analysis they are not yet available, although they are at different stages of the approval, pricing and reimbursement process.

In Spain, the SEOM and the Spanish Society of Pathology (SEAP) have published a clinical guideline to guide biomarker testing in patients with advanced NSCLC [1]. According to national and international recommendations for molecular diagnosis in advanced NSCLC patients, molecular testing of *EGFR* and *BRAF* mutations*, ALK* and *ROS1* rearrangements and PD-L1 expression are considered mandatory [1, 14]. *ROS1* rearrangement should be tested in patients with advanced stage (IIIB-IV) non-squamous NSCLC, regardless of its clinical characteristics and should not be tested in squamous cell carcinoma (except in the context of patients with no or low tobacco exposure and younger than 50 years) [1, 7, 14]. However, although the determination of *ROS1* is mandatory according to guidelines, real-world evidence obtained from Lung Cancer Biomarker Testing Registry (*LungPath*) show that *ROS1* fusions were not determined in almost half of the samples of patients with NSCLC (testing rate: 58.1%) [15]. According to the Thoracic Tumor Registry (TTR), an observational study also conducted in Spanish hospitals (up to the year 2018), showed even lower *ROS1* tests (testing rate [with FISH]: 11.6%]) [16]. This low rate of *ROS1* testing may be due to the low prevalence of *ROS1* rearrangements in patients with NSCLC that could discourage its determination in some centers, also conditioned by limited diagnostic and/or sampling resources [1, 6, 15].

Essentially, there are three methodological approaches to detecting *ROS1* rearrangements: immunohistochemistry (IHC), cytogenetic techniques (particularly fluorescent in situ hybridization [FISH], and molecular techniques such as real-time polymerase chain reaction (RT-PCR) or next-generation sequencing (NGS) [1, 17]. To determine *ROS1* translocation in clinical specimens, national and international guidelines recommend IHC as the screening method and confirmation of positive cases with another orthogonal method (cytogenetic or molecular) like FISH [1, 7]. FISH is often considered the gold-standard in the detection of *ROS1* rearrangement, although RT-PCR and NGS (DNA- or RNA-based) also show accurate results in most published studies [18–21].

Based on the clinical implications of *ROS1* fusion detection in NSCLC patients, it is crucial to accurately identify *ROS1* alterations while minimizing response time [1, 22]. The importance of testing for other biomarkers, such as *ALK*, has already been quantified in Spain by Nadal et al. [23], however, it has not been quantified for the determination of a less prevalent biomarker such as *ROS1*. For this reason, the main objective of this analysis was to quantify the clinical and economic impact of *ROS1* determination in patients with advanced NSCLC in Spain, comparing a *testing ROS1* strategy with sequentially testing *ROS1* in addition to *EGFR* and *ALK* versus a *no-testing ROS1* strategy.

## Methods

**Fig. 1:**
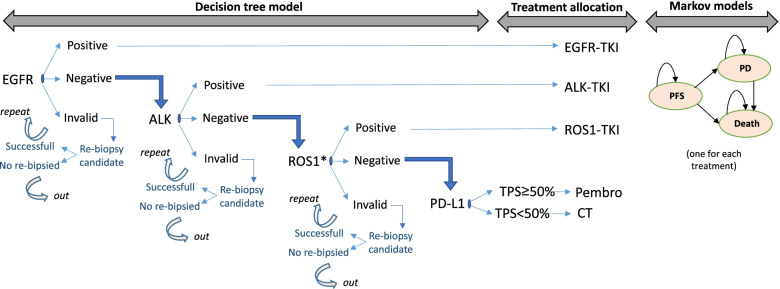
Joint model diagram combining a decision-tree model with Markov model. * ROS1 determined by IHC, FISH, reflex or NGS in ‘ROS1-testing’ scenario. Not determined in ‘no-ROS1-testing’ scenario. EGFR: epidermal growth factor receptor; ALK: anaplastic lymphoma kinase; ROS1: c-ros oncogene 1; PD-L1: programmed death-ligand 1; pembro: pembrolizumab monotherapy; CT: Chemotherapy; TKI: Tyrosine kinase inhibitors; PFS: progression-free survival; PD: progression disease

In line with the previous model developed by Nadal et al. [23], a joint model combining a decision-tree with Markov models was developed to determine long-term health results and associated costs of patients with NSCLC, but in this case by comparing a *testing ROS1* strategy by comparison against a *no-ROS1 testing* strategy in Spain, using Microsoft Excel (Fig. 1).

The decision-tree models comprise the diagnostic phase, where the sequential determination of *EGFR, ALK, ROS1* and PD-L1 were established. In case of a positive result for any of these biomarkers, the patient exits the model and receives the corresponding targeted treatment. In the model, in case of a negative result for *EGFR*, *ALK* and *ROS1* (defined as wild type [WT] patients), the level of PD-L1 expression is determined and the result is categorized as Tumor Proportion Score (TPS) ≥ 50% or TPS < 50%. This threshold of PD-L1 expression was defined based on the indication of pembrolizumab monotherapy for patients with high PD-L1 expression without oncogenic alterations in *EGFR* and *ALK*. At some point, the results for *EGFR*, *ALK* and *ROS1* can also be invalid, in which case patients will be direct candidates for re-biopsy. In the *no-ROS1 testing* strategy, the sequence shown in Fig. 1 excludes only *ROS1*, so in case of a negative result for *EGFR* and *ALK*, patients are directly considered as WT patients, and then the level of PD-L1 expression is determined.

Based on the determination results, a specific treatment is assigned (Fig. 1) and patients enter in the respective Markov model with different long-term clinical and economic outcomes. The Markov models are developed following an area under the curve structure with three health states: progression-free survival (PFS state), progressed-disease (PD state), and death state (absorbent state).

In line with the recommendations by the guidelines for the evaluation of health technologies in Spain, the duration of the Markov cycle was 1 month, the time horizon was 20-years (lifetime) and the discount rate for future costs and effects was 3% [24, 25].

The analysis was performed from the perspective of the Spanish National Health System (NHS), so only direct medical costs were considered (expressed in € 2021). The health consequences include life years (LY), progression-free life years (PF-LY) and quality-adjusted life years (QALYs).

The included parameters, the assumptions made as well as the clinical feasibility of the results were validated by a multidisciplinary group of oncologists and pathologists, who are also the authors of this article.

### Target population

The definition of the target population was similar to the one used in the previous model developed by Nadal et al. (2021) [23]. A hypothetical cohort of patients with advanced or metastatic NSCLC, who were ‘theoretical’ candidates for the molecular diagnosis, was initially estimated. Therefore, both patients with non-squamous histology and those with squamous NSCLC who were never smokers were considered, following the current clinical guidelines for molecular diagnosis in advanced NSCLC [1].

The estimation of the target population is shown in Table 1.

**Table 1:** Estimated target population

		%	Referencia	*n*
1	Patients with lung cancer in 2020		[26]	29,188
2	Patients with NSCLC	85.0%	[14]	24,810
3	Patients with stage IV NSCLC with sample available	54.5%	[27]	13,521
4	Patients with stage IV NSCLC non-squamous subtype	66.9%	[16]	9046
5	Patients with stage IV NSCLC squamous subtype	33.1%	[16]	4476
6	Patients with stage IV NSCLC squamous subtype, never smokers	11.9%	[28]	533
7	Candidate for molecular diagnosis (steps 4 + 6)			9579
8	Patients finally tested for *EGFR* (testing rate)	91.4%	[15]	**8755**

*NSCLC* Non-small cell lung cancer, *EGFR* Epidermal growth factor receptor

As shown in Fig. 1, the diagnostic sequence starts with *EGFR*, so of the theoretical patients estimated in Table 1, only those finally tested for *EGFR* entered the model.

### Decision-tree parameters

All the inputs that are used in the decision-tree sub-model are listed in Table 2.

**Table 2:** Main decision-tree inputs

	Input	Reference
Invalid results and positivity rate of selected biomarkers		
*EGFR* (% positive / % invalid)	13.6% / 1.70%	[15]
*ALK* (% positive / % invalid)	3.4% / 2.60%	[15]
*ROS1* (% positive / % invalid)	1.0% / 3.30%	[15]
PD-L1 (TPS ≥ 50%) (% positive / % invalid)	32.5% / 1.00%	Expert panel / [15]
Probability of re-biopsy		
Percentage of invalid results re-biopsied	33.3%	Expert panel
*ROS1* determination strategies		
IHC	10.0%	Expert panel
FISH	30.0%	Expert panel
REFLEX to FISH	55.0%	Expert panel
NGS	5.0%	Expert panel

*EGFR* Epidermal growth factor receptor, *ALK* Anaplastic lymphoma kinase, *ROS1* C-ros oncogene 1, *PD-L1* Programmed death-ligand 1, *IHC* Immunohistochemistry, *FISH* Fluorescent in situ hybridization, *NGS* Next-generation sequencing

As shown in Fig. 1, the results of the biomarker determinations can be informative (positive or negative) or invalid, mainly due to insufficient sample. The positivity rates for *EGFR, ALK* and *ROS1* determinations were obtained from *Lungpath* database while the PD-L1 positivity rate (considering TPS > 50% as the threshold for positivity) was agreed by the expert panel, given that the PD-L1 positivity rate obtained from *Lungpath* probably reflects a mixture of positivity rates with different thresholds depending on the center. Invalid rates for each biomarker were obtained from the *Lungpath* database. On the other hand, based on the experience of the experts, repeating invalid results does not usually give informative results, so it was assumed that invalid results would be direct candidates for re-biopsy (considered successful by experts in only 33.3% of cases). When re-biopsy is unsuccessful, patients receive doublet of chemotherapy if the molecular diagnosis of *EGFR* is unknown (due to an invalid result at the beginning of the sequential determination), or chemo-immunotherapy (the same received by patients with TPS < 50% or unknown PD-L1) if the invalid result was obtained for *ALK* or *ROS1* but the diagnosis of *EGFR* is known and negative.

The current distribution of *ROS1* determination techniques in Spain included in the model was obtained from the panel of experts, as the data provided by *Lungpath* reflects clinical practice in 2008 and does not correspond with current guideline recommendations where reflex to FISH is mandatory for *ROS1* determination. In the base case, the accuracy of the techniques is not taken into account, so that the specificity and sensitivity of all *ROS1* determination techniques were assumed to be 100%. For this reason, in the base case, the distribution of *ROS1* detection techniques only has an impact in terms of costs (not on health outcomes). However, an alternative scenario to the base case has been explored where the accuracy of the *ROS1* determination techniques is considered. The parameter values for IHC were obtained from the values of the IHC clones from the ROSING study [21] and the distribution of clones agreed by the experts (70% SP384, 30% D4D6). The resulting sensitivity and specificity of IHC was 90.9 and 99.0%, respectively. For FISH, the specificity and sensitivity values agreed by the experts were 99 and 95%, respectively, and for NGS, the values of both parameters were assumed to be 100% despite that in rea-life testing the sensitivity and sensitivity of NGS would not be 100%.

The specific costs of the decision-tree were the cost of re-biopsy and the costs of the tests used for molecular diagnosis. For the re-biopsy, a cost of € 385.41 was considered. It was calculated by weighting the distribution of the type of biopsy performed (biopsy, cytology, blood: 65, 30, 5%, respectively) as reported by experts and the cost of each of the biopsy techniques (€ 555.70, € 58.70, € 131.84, respectively) [29]. The costs of the tests were agreed by the expert panel, taking into consideration the market price: € 70 for IHC; € 110 for FISH; € 455 for NGS; € 120 for the *EGFR* test; € 76 for the *ALK* test; and € 70 for the PD-L1 test [29].

As described in Table 3, thirteen treatments and inclusion in clinical trials were included in the analysis, and for each, costs and long-term outcomes are quantified using a specific Markov model. The expert panel established the distribution of all the most common first-line treatments in Spain for each molecular profile (Table 3).

**Table 3:** Distribution of treatments according to molecular diagnosis

Molecular diagnosis	Treatment	Distribution
*ALK*+	Alectinib	95%
	Crizotinib	5%
EFGR+	Erlotinib	3,3%
	Gefitinib	6,7%
	Afatinib	11.7%
	Osimertinib	76.3%
	Dacomitinib	2%
*ROS1*+	Crizotinib	95% (40%^a^)
	Clinical trials	5%
	Entrectinib	0% (55%^a^)
WT		
TPS ≥50%	Pembrolizumab monotherapy	90%
	Cisp+pmtrx	5%
	Clinical trials	5%
TPS < 50%	Cisp+pmtrx	25%
	Carb+paclitx+beva	5%
	Cisp+pmtrx+pembrolizumab	60%
	Carb+ paclitx +beva+atezolizumab	10%

*EGFR* Epidermal growth factor receptor, *ALK* Anaplastic lymphoma kinase, *ROS1* C-ros oncogene 1, *WT* Wild-type, *TPS* Tumour proportion score, *Cisp* Cisplatin, *Carb* Carboplatin, *pmtrx* Pemetrexed, *paclitx* Paclitaxel, *beva* Bevacizumab

^a^Distribution considered in the alternative scenario in which entrectinib is a treatment alternative in ROS1-positive patients

An alternative scenario to the base case has been explored considering a potential treatment distribution scenario where *ROS1*-positive patients may also receive entrectinib, as although it is not yet commercially available for these patients in Spain, experts estimate that its use will increase in the future and could have some impact on the model results, unlike other upcoming ALK-targeted therapies that would have negligible effects on the results (Table 3).

Depending on the molecular diagnosis result, patients were assigned to a specific treatment and entered the respective Markov model developed, in which efficacy and costs associated with each treatment were considered.

### Markov model parameters

To establish the transition of the hypothetical cohort between the health states of the Markov models and given that the time horizon of the analysis (lifetime) is longer than the observation periods of the clinical trials, it is necessary to extrapolate the Kaplan-Meier curves of PFS and OS to the long term. In the absence of individualized data for all treatments to explore different parametric distributions, the panel of experts assumed, in line with the previous model developed, the exponential models based on the median PFS and OS reported in the respective studies [23].

Median PFS and OS for *ALK* targeted therapies (alectinib, crizotinib) were obtained from the recent update of the ALEX study [30], except the median OS in the alectinib group was not reached and extrapolation curves were obtained from the alectinib cost-effectiveness model (data on file). Median PFS and OS for *EGFR*-targeted therapies were obtained from FLAURA study (assuming the same efficacy for afatinib as for erlotinib and gefitinib) [31, 32] and from ARCHER 1050 study for dacomitinib [33]. The PROFILE 1001 study was used for the median PFS and OS of crizotinib as a targeted therapy for *ROS1* [10], and for the median PFS and OS of entrectinib, the entrectinib cost-effectiveness model (data on file) was used, given that in its STARTRK-2 study, the median OS has not yet been reached. For WT patients treated with pembrolizumab in monotherapy, median PFS and OS were obtained from KEYNOTE-024 [34, 35] and for WT patients treated with pembrolizumab in combination and cisplatin + pemetrexed, the medians of the parameters were obtained from the KEYNOTE-189 study (comparator and control arm, respectively) [36]. For WT patients with TPS < 50%, median PFS and OS for the remaining two treatment strategies considered in the model were obtained from Sandler et al. (2006) [37] and IMpower150 [38]. For patients entering a clinical trial, a small percentage of *ROS1*-positive and WT with TPS ≥50% patients, the experts assumed extrapolation with the longest medians of the corresponding therapeutic target (medians PFS and OS of crizotinib and pembrolizumab monotherapy, respectively).

In the alternative scenario considering the accuracy of the *ROS1* determination techniques it was necessary to establish the clinical consequences of the false positive (FP) results for *ROS1*. In this regard, the expert panel agreed to keep the same assumptions made in the previous model developed. Thus, it was considered that most patients will have shown progression at the first follow-up visit and that all of them will have progressed at the second visit, assuming a median PFS of 2 months (considering that no patient exceeds 6 months of treatment -stop rule-) and a median OS of 18 months [23].

The costs of the Markov models included drug acquisition costs (first line and subsequent treatments) [see Additional file 1] and its associated cost of administration in case of intravenous drugs (€ 211) [29].

For the acquisition costs, all drug costs are expressed as the ex-factory price considering the corresponding deductions according to RDL 08/2010 [39, 40] where appropriate. For drugs where the dose depends on the patient’s characteristics, the same demographic characteristics of the hypothetical cohort from the previous model were assumed, that is, a mean body surface area of 1.81 and a mean weight of 72.885 kg [41]. The vial sharing was assumed for intravenous treatment in line also with previous model. For entrectinib, which was not yet priced at the time of analysis, an ex-factory price 10% higher than crizotinib was assumed. Clinical trials are assumed to have no cost to the Spanish NHS.

The efficacy of the treatments (in terms of median PFS and median OS) and the costs associated with them are shown in an additional file [see Additional file 1].

Concerning the costs of subsequent treatments administered once patients progress to first-line treatment, only the costs of second-line treatments were considered in order to simplify the model. Both the proportion of patients who would receive active second-line treatment and those who would receive best supportive care (BSC), as well as the distribution of the most representative second-line treatments (depending on the first-line received) was established by the experts. Median PFS of all subsequent treatments were obtained from the literature [11, 42–46]. The parameters related to the second-line treatments are shown in Table 4.

**Table 4:** Definition of subsequent (second-line) treatments

Molecular diagnosis	First-line treatment	Second-line treatment			
		Active treatment		Non-treatment	
		%	Treatment	%	Treatment
*ALK*+	Alectinib	80%^a^	PbCT/lorlatinib	20%^a^	BSC
	Crizotinib	80%^a^	Alectinib	20%^a^	BSC
*EGFR*+	Osimertinib	70%^a^	PbCT	30%^a^	BSC
	Erlotinib/Gefitinib/Afatinib/Dacomitinib	70%^a^	Osimertinib	30%^a^	BSC
*ROS1*+	Crizotinib/Clinical trial/Entrectinib	80%^a^	PbCT	20%^a^	BSC
WT					
TPS ≥50%	Pembrolizumab/Cisp+pmtrx/Clinical trial	60.80%^b^	PbCT	39.20%^b^	BSC
TPS < 50%	Cisp+pemetrexed	46.60%^c^	immunotherapies/doce+nintedanib	53.40%^c^	BSC
	carb+paclitx+beva	46.60%^c^	immunotherapies/doce+nintedanib	53.40%^c^	BSC
	cisp+pmtrx+pembrolizumab	30.50%^c^	Doce+nintedanib	69.50%^c^	BSC
	carb+paclitx+beva+atezolizumab	30.50%^c^	Doce+nintedanib	69.50%^c^	BSC

For the grouping of PbCT/lorlatinib and immunotherapies/doce+nintedanib an arithmetic mean was considered

^a^expert panel

^b^ [47]

^c^ [48]

*EGFR* Epidermal growth factor receptor, *ALK* Anaplastic lymphoma kinase, *ROS1* C-ros oncogene 1, *WT* wild-type, *Carb* Carboplatin; pmtrx: pemetrexed, *paclitx* Paclitaxel, *beva* Bevacizumab, *PbCT* Platinum-based chemotherapy, *doce* Docetaxel, *BSC* Best supportive care

Regarding the utility values included, the experts decided to use the same values as those applied in the previously developed model (0.814 for the PFS state and 0.725 and 0.470 for the PD state with and without active treatment, respectively) [23, 49].

### Sensitivity analysis

The variables used in the model have some uncertainty. To assess and determine the robustness of the results obtained, both deterministic (alternative scenarios to the base case and univariate analysis) and probabilistic sensitivity analyses were performed.

Alternatively to the main analysis (base case), two scenarios (described throughout the article) were also explored within the sensitivity analysis:

- Considering a potential scenario in which entrectinib (not present in the base case scenario) is a treatment alternative in *ROS1* positive patients (Table 3).
- Considering the accuracy of the different techniques for *ROS1* determination, where the specificity and sensitivity parameters of the different *ROS1* determination techniques are included (detailed in 2.3 Decision-tree parameters section).

In the univariate deterministic analysis (one-way sensitivity analysis), some variables of the model were individually modified, depending on the degree of uncertainty associated with the variable, by 10% or 20% with respect to the base case value.

In the probabilistic sensitivity analysis (PSA), in line with recommendations in the literature [50], 1000 simulations were run by Monte-Carlo method simultaneously modifying all parameters with an established distribution. The biomarker prevalence variables, body weight and body surface area and the probability of re-biopsy in case of an invalid result, were modified by a normal distribution, utility values by a beta distribution, and unit costs were modified following a gamma distribution.

## Results

### Main analysis (base case)

**Fig. 2:**
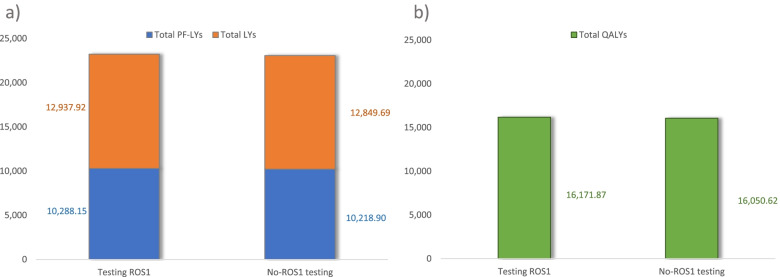
Long-term health outcomes associated to *testing*-ROS1 and to *no-testing* ROS1 strategies, base case. **a** Health outcomes expressed in total PF-LYs and total LYs; **b** Health outcomes expressed in total QALYs. ROS1: c-ros oncogene 1; PF:Progression-free life years; LY: life years; QALY: quality-adjusted life years

**Fig. 3:**
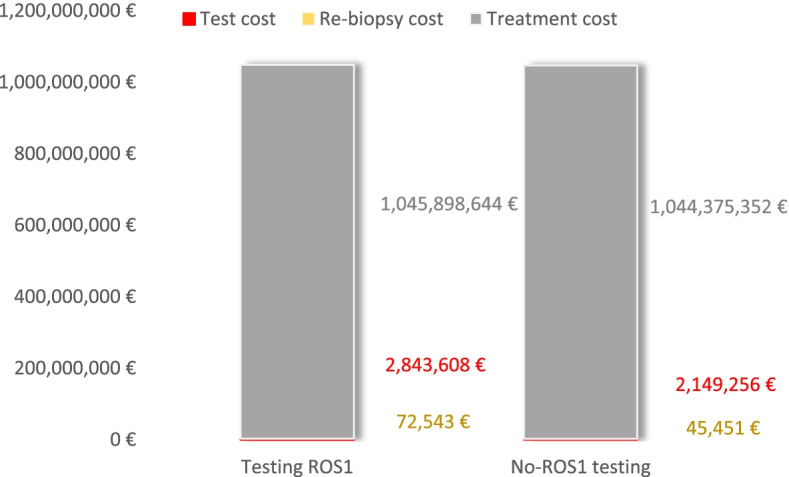
Long term cost outcomes associated to *testing*-ROS1 and to *no-testing* ROS1 strategies, base case. ROS1: c-ros oncogene 1

The results of the base case are reported in Table 5 and are shown graphically in Fig. 2 (health outcomes) and Fig. 3 (cost outcomes).

**Table 5:** Base case results: cost-effectiveness of testing *ROS1* strategy vs. no-testing *ROS1*

	*Testing ROS1*	*No-testing ROS1*	Difference
*Cost of testing*	*€ 2,843,608*	*€ 2,149,256*	*€ + 694,352*
*Cost of re-biopsy*	*€ 72,543*	*€ 45,451*	*€ + 27,093*
*Cost of treatment*	*€ 1,045,898,644*	*€ 1,044,375,352*	*€ + 1,523,292*
Total costs	**€ 1,048,814,795**	**€ 1,046,570,058**	**€ + 2,244,737**
PF-LYs	*10,288.15*	*10,218.90*	*+ 69.25*
LYs	*23,226.07*	*23,068.58*	*+ 157.49*
QALYs	*16,171.87*	*16,050.62*	*+ 121.25*
ICER (€/LY gained)			**€ 14,254/LY**
ICUR (€/QALY gained)			**€ 18,514/QALY**

*ROS1* C-ros oncogene 1, *PF* Progression-free, *LY* Life years, *QALY* Quality-adjusted life years, *ICER* Incremental cost-effectiveness ratio, *ICUR* Incremental cost-utility ratio

In the defined target population, the strategy of *testing-ROS1* in patients with advanced NSCLC provided a gain of 121.25 QALYs compared with the *no-testing ROS1* strategy over a 20-year time horizon. *Testing ROS1* strategy in these patients also entailed higher costs, including those of the tests themselves and the re-biopsies, but mainly due to the cost of targeted treatments. The comparison of costs and health outcomes through the incremental cost-utility ratio (ICUR), shows that the *testing ROS1* strategy in Spain is cost-effective (€ 18,514/QALYs), as it was below the cost-effectiveness thresholds commonly considered in Spain [51, 52].

### Sensitivity analysis

The cost and health results of the alternative scenarios are in line with those of the base case (Table 5). The modifications made in both analyses from the base case affect only the costs and health outcomes of the *testing ROS1* strategy (*no testing ROS1* strategy remained the same).

In the alternative scenario considering a potential future scenario in which entrectinib is available in *ROS1*-positive patients, the *testing ROS1* vs. *no-testing ROS1* strategy remains cost-effective, with an ICUR ratio of € 17,652/QALYs (slightly lower than in the base case). According to the results, although the inclusion of entrectinib in the treatment of *ROS1*-positive patients results in a slight increase in the treatment costs of testing *ROS1* strategy compared to the corresponding base case strategy (€ 239,199 more € than in base case vs. € 1,044,375,352 in base case), it also results in an increase in QALYs gained (19.47 QALYs more than in base case; 140.72 QALYs in alternative scenario vs. 121.25 QALYs in base case).

In the alternative scenario that considers the accuracy of the techniques the *testing ROS1* vs. *no-testing ROS1* strategy is dominant, generating more QALYs at a lower cost. Although in this analysis there is a slight decrease in QALYs gained from the *testing ROS1* strategy compared to the corresponding base case strategy (23.1 QALYS less than in base case; 98.15 QALYs in alternative scenario vs. 121.25 QALYs in base case), there is a more significant decrease in the costs of the strategy compared to the base case (€ 8,257,604 less than in base case vs. € 1,048,814,795 in base case), due mainly to the treatment costs.

**Fig. 4:**
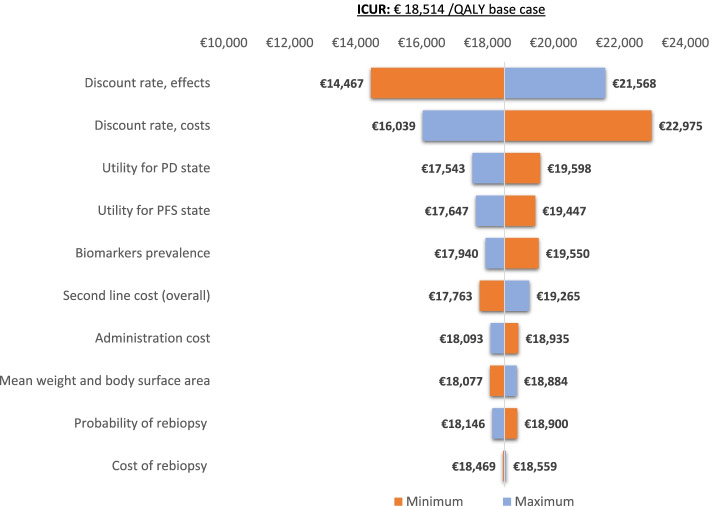
Tornado diagram representing the results of the univariate analysis. ICUR: incremental cost-utility ratio; QALY: quality-adjusted life years; PD: progressed-disease; PFS: progression-free survival

The results of the univariate analysis are represented by a tornado diagram in Fig. 4, showing how the variations of each variable analyzed modify the ICUR of the base case (€ 18,514 /QALYs). Discount rate (for both cost and effects), followed by utilities show the greatest impact on the ICUR of the base case.

**Fig. 5:**
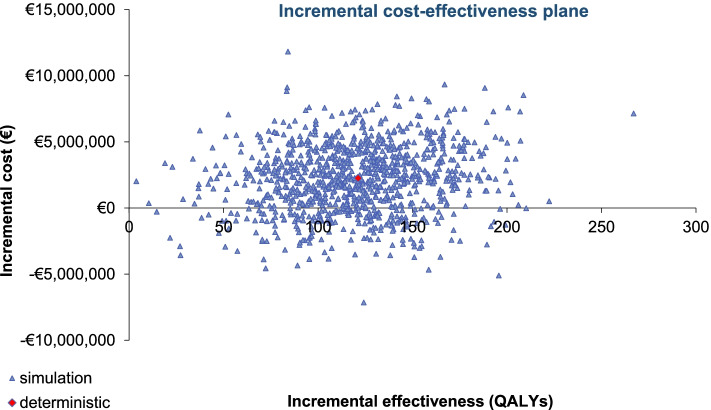
PSA results of 1000 simulations provided by incremental cost-effectiveness plane. QALY: quality-adjusted life years

In the PSA, the means obtained from the 1000 simulations (€ + 2,209,967 and 18,456 QALYs gained with respect to *no-testing ROS1* strategy) are in line with the deterministic results in Table 5. The graphical representation of the 1000 simulations is provided by an incremental cost-effectiveness plane (Fig. 5).

## Discussion

The effectiveness of targeted therapies with tyrosine kinase inhibitors in NSCLC depends on the accurate determination of the genomic status of tumor cells. For this reason, specific biomarker testing by molecular methods is widely recommended by guidelines in patients with advanced NSCLC [7, 53].

In particular, the identification of *ROS1* rearrangement in NSCLC patients is mandatory to permit targeted therapy with specific inhibitors, demonstrating an improved overall survival when compared with conventional chemotherapy [1, 6, 7]. Treatment of *ROS1*-positive NSCLC patients with crizotinib has demonstrated a clinically significant benefit (with more than 19 and 51 months of PFS and OS, respectively), and the benefit of other targeted therapies, such as entrectinib are being studied [1, 10].

The availability of specific inhibitors of *ROS1* and their clinical benefit should lead to simultaneous testing of ROS1 rearrangement with other recurrent biomarkers in NSCLC (e.g EGFR and ALK) in all advanced stage never/light smokers with squamous cell carcinoma and non-squamous NSCLC [6]. However, according to the analysis carried out by Salas et al. (2021) [15] in Spain in 2018, despite the relatively high testing rate reported in *EGFR* and *ALK* in NSCLC (91.4 and 80.1%, respectively), the real-world evidence obtained from the *LungPath* registry demonstrates that *ROS1* and PD-L1 were not determined in a significant portion of patients (56.2 and 58.1%, respectively) [15].

.Given the incremental importance of *ROS1* testing in guiding the treatment of patients with NSCLC and the fact that *ROS1* is an under-analyzed biomarker in comparison to *ALK* or *EGFR* in Spain [15], the main objective of our study was to assess if *testing ROS1* is a cost-effectiveness strategy in Spain and also to raise the awareness of *testing ROS1* according the clinical guidelines.

Our study is the first one that evaluate the efficiency of *ROS1* determination in the management of patients with advanced NSCLC in Spain using an approach that integrates molecular diagnosis with subsequent pharmacological treatment.

Markov models allowed the calculation of long-term costs, LYs and QALYs for each treatment assigned according to the molecular diagnosis. In addition, the sequential decision-tree allowed comparison of the *ROS1 testing* strategy vs. *no ROS1 testing* strategy in Spain (*EGFR*, *ALK* and PD-L1 expression were tested in both strategies) and establish the subsequent treatment allocation according to the molecular diagnosis.

Thus, the ICUR obtained (for the base case and sensitivity analyses) demonstrates that the *testing ROS1* strategy in patients with advanced NSCLC is a cost-effective strategy as it is below the cost-effectiveness thresholds usually considered in our country [51, 52].

Given that soon entrectinib will be available for *ROS1*-positive patients as a first-line targeted therapy (currently the only existing treatment is crizotinib), the sensitivity analysis also included entrectinib as a possible treatment for these patients. In this alternative scenario, the strategy of testing *ROS1* vs. no testing is also remained as a cost-effective strategy (with a lower ICUR than in the base case). On the other hand, in a scenario that could be closer to real-world practice than the base case, which considers the accuracy of molecular diagnostic techniques, *ROS1 testing* strategy is shown to be dominant over *no-ROS1 testing*, with an increase in QALYs at a lower cost. However, given the uncertainty associated with the specificity and sensitivity parameters of the determination techniques, the results of this alternative scenario must be interpreted with caution.

Despite the low prevalence of *ROS1* rearrangements in patients with advanced NSCLC (~ 1%), these results confirm that *ROS1 testing* is of crucial interest for the treatment of patients with advanced NSCLC [1, 6].

Prior to this joint model, the same group of experts developed a similar one to determine the efficiency of management of patients with advanced NSCLC in Spain but focusing on *ALK* detection [23]. Although both joint models have in common the integration of molecular diagnosis and subsequent treatment depending on the molecular results, they have also some methodological differences. First, the current work analyses the determination of *ROS1* within the *EGFR, ALK, ROS1* and PD-L1 sequence. In contrast, the previous study focused exclusively on the determination of *ALK*, and although it included other biomarkers, these were analyzed following *ALK* testing. In addition, the previous work considered the different *ALK* testing techniques and their accuracy (specificity and sensitivity), as the objective was to evaluate the current *ALK* testing scenario. In the present analysis, we focused on whether or not *ROS1* was tested, so the accuracy of the *ROS1* techniques was assessed only as an alternative scenario. According to the results of both studies, the strategies of testing *ALK* and *ROS1* vs. no testing, not only generate more than 3000 QALYs and 120 QALYs, respectively, and remain cost-effective strategies in the Spanish context (€ 10,142 /QALY and € 18.514 /QALY, respectively) [23].

No other studies that analyze specifically the implications of *ROS1* determination in our European context have been identified. A recent Canadian study has compared different diagnostic strategies for ROS1 testing using a decision model, obtaining that reflex IHC screening with FISH confirmation of positive cases yielded the best results for turnaround time, true-positives detection rate, and costs [54]. In a broader context, recent studies have reported on the factors that most affect the biomarker determination value such as that conducted by Safonov et al. (2016) in the United States, and also the cost-effectiveness of different NSCLC biomarker testing strategies including *ROS1* among them, as the study carried out by Schluckebier et al. (2020) from the Brazilian health system perspective [55, 56].

Like all theoretical models, our study has several limitations. Firstly, the complexity of clinical practice cannot be fully captured by pharmacoeconomic models which are designs with a certain degree of intrinsic structural rigidity. In this particular case, capturing and reproducing all phases of pathological and molecular diagnostics is complicated by the fact that, according to the studies identified, they have a high degree of complexity and variation between centers. In line with the previous study focusing on *ALK* determination, to simplify the model the pre-analytical phase (e.g. response time from diagnosis to initiation of treatment) was not included as there is no evidence that it influences the results.

Certain assumptions have been made to model the treatment of invalid results and the probability of re-biopsy in the diagnostic phase. To simplify the model, it was assumed that after an invalid result there is no repetition of the technique using the same sample, but re-biopsy is considered directly. This assumption was made because, according to expert opinion, repeat testing on the same sample is rarely satisfactory and recoverable tests accounts for less than 10%. Regarding the success rate of re-biopsy, no reference was identified in the literature that according to the experts is in line with their clinical experience, where in 30–35% of cases an optimal re-biopsy specimen is achieved. Thirdly, since the main objective of the analysis was to determine the significance of *ROS1* determination and not so much the validation of the testing techniques, a specificity and sensitivity of the techniques of 100% was assumed and validated by experts, which is far from real clinical practice. However, this limitation was evaluated in one of the alternative scenarios.

Fourth, Markov models have some limitations when reproducing at a long term the clinical management of NSCLC patients. On the one hand, as in all cost-effectiveness models where long-time horizons are used, extrapolations are necessary with the associated limitations. On the other hand, as described above, in the absence of individualized data for all treatments included in the analysis, it was assumed the use of exponential models for all treatments, as conducted in Nadal. et al. (2021) [23]. In the same way, no drug-specific utilities were found for all the treatments included in the model. Therefore, utility values associated with the patient’s condition (PFS, PD on-treatment and PD without active treatment) regardless of the treatment received were used, in line with previous economic evaluations [23, 49]. As our analysis focuses on molecular diagnosis and subsequent first-line treatment of NSCLC patients, the second-line treatment included in the analysis was entered into the model as a one-off cost and only shows its influence in economic terms.

Fifthly, and in relation to costs, it is difficult to establish the real price of the diagnostic tests and although these were validated by experts, there are large variations between centers and in many cases, as they are not reimbursed by the Spanish NHS, the cost of the tests is mainly assumed by the pharmaceutical companies and research funds in the vast majority of Spanish regions. On the other hand, the fact that the model assumed a sequential determination and that it focused on the determination of *ROS1* did not allow us to capture the benefits of using NGS as a technique to evaluate multiple biomarkers simultaneously. An economic assessment of the use of NGS instead of sequential *ROS1* testing in addition to *EGFR* and *ALK* will require a specifically designed model and is beyond the scope of this study.

Despite these limitations and the associated uncertainty, sensitivity analyses were carried out which confirmed the robustness of the assumptions and results obtained. It should also be noted that the panel of experts validated all the assumptions, parameters considered, and results obtained.

## Conclusions

In conclusion, despite the low prevalence of *ROS1* rearrangements in advanced NSCLC patients, our analysis shows that *testing ROS1* in these patients in Spain is a strategy that would increase and improve patients’ lives and that it is a cost-effective strategy for the Spanish NHS. For this reason, this analysis should encourage that not even a single *ROS1*-positive patient goes undiagnosed.

## Supplementary Information

**Additional file 1: Table 1.** Efficacy (median PFS and median OS) and costs associated with each first-line treatments.

## Data Availability

Qualified researchers may request access to individual patient level data through the clinical study data request platform (https://vivli.org/). Further details on Roche’s criteria for eligible studies are available here (https://vivli.org/members/ourmembers/). For further details on Roche’s Global Policy on the Sharing of Clinical Information and how to request access to related clinical study documents, see here (https://www.roche.com/research_and_development/who_we_are_how_we_work/clinical_trials/our_commitment_to_data_sharing.htm).

## Abbreviations

ALK: Anaplastic lymphoma kinase
Beva: Bevacizumab
BSC: Best supportive care
Carb: Carboplatin
Cisp: Cisplatin
CT: Chemotherapy
Doce: Docetaxel
EGFR: Epidermal growth factor receptor
FISH: Fluorescent in situ hybridization
FP: False positive
ICER: Incremental cost-effectiveness ratio
ICUR: Incremental cost-utility ratio
IHC: Immunohistochemistry
LC: Lung cancer
LF: Life years
LungPath: Lung cancer biomarker testing registry
NGS: Next-generation sequencing
NHS: National health system
NSCLC: Non-small cell lung cancer
Paclitx: Paclitaxel
PbCT: Platinum-based chemotherapy
PD: Progression disease
PD-L1: Programmed death-ligand 1
PF-LY: Progression-free life years
PFS: Progression-free survival
Pmtrx: Pemetrexed
PSA: Probabilistic sensitivity analysis
QALYs: Quality-adjusted life years
ROS1: C-ros oncogene 1
RT-PCR: Real-time polymerase chain reaction
SEAP: Spanish society of pathology
SEOM: Spanish society of medical oncology
TKI: Tyrosine kinase inhibitors
TPS: Tumor proportion score
TTR: Thoracic tumor registry
WT: Wild type
