# Supplementary material for: Clinical and economic impact of ‘ROS1-testing’ strategy compared to a ‘no-ROS1-testing’ strategy in advanced NSCLC in Spain
Source: BMC Cancer. 2022 Mar 19;22:292. doi: 10.1186/s12885-022-09397-4 (PMC8933896; doi:10.1186/s12885-022-09397-4)
Supplement: Supplementary file 1 — Additional file 1: Table 1. Efficacy (median PFS and median OS) and costs associated with each first-line treatments. [file 12885_2022_9397_MOESM1_ESM.docx]

# Additional file 1

**Table Additional file 1**: Efficacy (median PFS and median OS) and costs associated with each first-line treatments

|  | **Treatments** | **Progression-free survival** | | | **Overall survival** | | | **Cost/week (€)***‡* | |
| --- | --- | --- | --- | --- | --- | --- | --- | --- | --- |
|  |  | **mPFS** | **Reference** | **mOS** | | **Reference** |  | |  |
| ALK+ | Alectinib | 34.8 | (30) | n.r | | Data on file | € 1.298 | |  |
|  | Crizotinib | 10.9 | (30) | 57.4 | | (30) | € 1.129 | |  |
| EGFR+ | Erlotinib | 9.7 | (31,32) | 31.8 | | (31,32) | € 275 | |  |
|  | Gefitinib | 9.7 | (31,32) | 31.8 | | (31,32) | € 286 | |  |
|  | Afatinib | 9.7 | Assumption* | 31.8 | | (31,32) | € 441 | |  |
|  | Osimertinib | 17.7 | (31,32) | 38.6 | | (31,32) | € 1.309 | |  |
|  | Dacomitinib | 14.7 | (33) | 34.10 | | (33) | € 583 | |  |
| ROS | Crizotinib | 19.3 | (10) | 51.4 | | (10) | € 1.129 | |  |
|  | Entrectinib | 19.3 | Assumption | 51.4 | | Assumption | € 1.242 | |  |
| WT | Pembro monotherapy | 10.3 | (34,35) | 26.3 | | (34,35) | € 6.597 *^¥^* | |  |
|  | Cisplatin+pemetrexed | 4.9 | (36) | 10.7 | | (36) | Cisp*^¥^*: € 30 (4 cycles)  Pmtrx*^¥^* : € 2.009 | |  |
|  | Cisplatin+pemetrexed+  pembrolizumab | 9.0 | (36) | 22.0 | | (36) | Cisp*^¥^*: 30 € (4 cycles)  Pmtrx*^¥^* : 2.009 €  Pembro*^¥^*: 6.597 € | |  |
|  | Carboplatin+paclitaxel+  bevacizumab | 6.2 | (37) | 12.3 | | (37) | Carb+ pcltx*^¥^*: 573 € (4 cycles)  Beva*^¥^*: 3.218 € | |  |
|  | Carboplatin+paclitaxel+  bevacizumab+atezolizumab | 8.3 | (38) | 19.2 | | (38) | Carb+ pcltx*^¥^*: 573 € (4 cycles)  Beva*^¥^*: 3.218 €  Atezo*^¥^*: 4.152 € | |  |

*EGFR: epidermal growth factor receptor; ALK: anaplastic lymphoma kinase; ROS1: c-ros oncogene 1; WT: wild-type; Carb: carboplatin; pmtrx: pemetrexed; pcltx; paclitaxel; beva: bevacizumab; mPFS: median Progression free survival; mOS: median overall survival; n.r.: not reached.*

** assumed the same efficacy for afatinib as for erlotinib and gefitinib; ¥ costs are expressed as cost each 3 weeks; ‡ drug acquisition costs were obtained from (39)*
